# Strategically targeting MYC in cancer

**DOI:** 10.12688/f1000research.7879.1

**Published:** 2016-03-29

**Authors:** Valeriya Posternak, Michael D. Cole

**Affiliations:** 1 Department of Pharmacology and Toxicology, Geisel School of Medicine at Dartmouth, Norris Cotton Cancer Center, Lebanon, NH, USA; 2 Department of Genetics, Geisel School of Medicine at Dartmouth, Norris Cotton Cancer Center, Lebanon, NH, USA

**Keywords:** MYC, cancer, transcriptional control, cancer therapy, inhibitors

## Abstract

MYC is a major driver of cancer cell growth and mediates a transcriptional program spanning cell growth, the cell cycle, metabolism, and cell survival. Many efforts have been made to deliberately target MYC for cancer therapy. A variety of compounds have been generated to inhibit MYC function or stability, either directly or indirectly. The most direct inhibitors target the interaction between MYC and MAX, which is required for DNA binding. Unfortunately, these compounds do not have the desired pharmacokinetics and pharmacodynamics for *in vivo* application. Recent studies report the indirect inhibition of MYC through the development of two compounds, JQ1 and THZ1, which target factors involved in unique stages of transcription. These compounds appear to have significant therapeutic value for cancers with high levels of MYC, although some effects are MYC-independent. These approaches serve as a foundation for developing novel compounds to pharmacologically target MYC-driven cancers.

## Introduction

The MYC protein plays a crucial role in a variety of cellular processes, including cell proliferation and differentiation, cell cycle progression, metabolism, and apoptosis ^1, 2^. MYC is a pleiotropic transcription factor that regulates a variety of functions by promoting activation or repression of genes on a global scale ^3– 5^. As a transcription factor, MYC heterodimerizes with MAX and directly binds to a consensus sequence on DNA, CACGTG ^6^. MYC-mediated transcriptional activation involves an interaction between MYC and a nuclear cofactor, transformation/transcription domain-associated protein (TRRAP), through a conserved domain on MYC, MYC Box II (MBII) ^7^. TRRAP is in complex with histone acetyltransferases that acetylate histones around gene promoters, inducing an open chromatin conformation, making it possible for RNA polymerase II (RNA Pol II) recruitment and productive transcription ^8, 9^.

**Figure 1.**
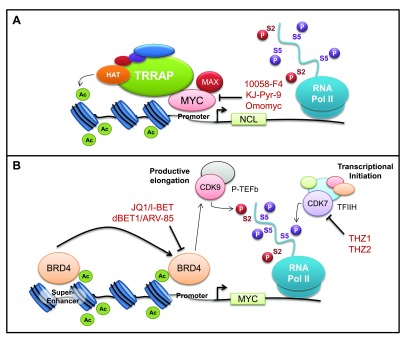
Direct and indirect inhibition of MYC. ( **A**) Targeting the MYC/MAX interface by using 10058-F4, KJ-Pyr-9, or Omomyc inhibits binding to DNA and the MYC transcriptional pathway. ( **B**) Indirect targeting of MYC expression through inhibition of CDK7 or BRD4, key factors involved in transcriptional initiation and elongation, using JQ1/dBET1 or THZ1/THZ2, respectively. Targeting CDK7 or BRD4 results in specific downregulation of MYC protein expression.

MYC expression is tightly regulated under normal circumstances and is increased in response to extracellular stimuli, such as growth factors ^10, 11^. Chromosomal translocation, gene amplification, and mutations in signaling pathways promote MYC overexpression independently of growth factor stimulation, which leads to unrestrained cell proliferation and tumorigenesis ^12^. MYC is deregulated in approximately 70% of human cancers ^3^, and many studies have observed that MYC inhibition can result in tumor regression and cell differentiation in a host- and cell-dependent manner ^13^. Widespread activation of MYC in a range of tumors and the reversibility of MYC-induced tumorigenesis have made MYC an appealing target for cancer therapy. However, MYC lacks innate enzymatic function and small-molecule interactions that facilitate most pharmacological strategies. Furthermore, as a transcription factor, MYC is localized in the nucleus and hence is inaccessible to any antibody-based therapies. For these reasons, MYC is widely considered ‘undruggable’, a frustrating limitation for such a well-established driver of cancer. Nevertheless, numerous strategies have been employed to target MYC at various stages of biological and pathological development, and there have been significant advances in understanding the MYC dependence of cancer and developing novel approaches to targeting MYC activity in the past five years ( Figure 1). To date, directly targeting MYC’s interaction with MAX by using compounds like 10058-F4 has proven unsuccessful *in vivo*, although a biological agent, Omomyc, has proven informative. However, a new library screen resulted in the identification of a potent *in vivo* MYC-MAX inhibitor, KJ-Pyr-9, that has some efficacy ^14^. More recent studies have demonstrated that indirect approaches using compounds designed to inhibit key factors involved in transcriptional initiation and elongation seem selective for the MYC oncogenic pathway ^15– 18^. These new developments in therapeutic targeting of MYC in cancer have broad implications in a challenging field where inhibition of MYC has been shown to result in tumor regression but has proven problematic to execute because of difficulties in delivery or specificity.

## Targeting the MYC and MAX interface

Since dimerization with MAX is essential for MYC DNA-binding activity ^19^, disruption of the MYC/MAX interaction by using small molecules is an obvious strategy of targeting MYC functionality. A number of selective low molecular weight inhibitors that disrupt the interaction between MYC and MAX have been developed ^20^. One of these is 10058-F4, a molecule that prevents heterodimerization and is capable of penetrating cells with low non-specific toxicity ^21, 22^. The compound has demonstrated the ability to inhibit mammalian cell growth, cell cycle progression, and expression of MYC target genes *in vitro*. A number of studies have reported that short-term pharmacological inhibition of MYC using 10058-F4 or more potent analogs leads to tumor regression *in vivo*. More recently, KJ-Pyr-9, a compound identified in a pyridine library screen, was identified as a potent inhibitor of the MYC/MAX interaction and it displays the correct pharmacokinetic properties necessary for *in vivo* administration ^14^. Although these compounds have shown specificity for the MYC/MAX interaction, targeting a bHLH-LZ domain is inherently inefficient and potentially non-specific since many other proteins contain these motifs. Nevertheless, 10058-F4 and KJ-Pyr-9 appear to have differential efficacy *in vivo*, depending on tumor type, differential metabolism of the compounds, and tumor model ^14, 23– 25^. Taken together, these data suggest that direct inhibition of MYC through disruption of the MYC/MAX interaction is promising but requires further experimentation to establish specificity and efficiency in humans.

A second strategy to inhibit MYC/MAX dimerization is Omomyc, a mutant basic helix-loop-helix domain that acts as a potent dominant negative molecule by sequestering MYC and preventing its binding to MAX and DNA ^26, 27^. Under normal circumstances, MYC is unable to homodimerize, but Omomyc is a MYC homolog that contains four amino acid substitutions augmenting homodimerization and non-functional heterodimerization with MYC. Although Omomyc cannot penetrate human tumors and hence is ineffective as a cancer therapeutic, it has proven useful to explore the consequences of MYC inhibition *in vivo*. Omomyc can stimulate MYC-induced apoptosis of NIH3T3 cells in a MYC-dependent manner *in vitro* and of MYC-overexpressing tumor cells in a mouse model of K-Ras-driven lung adenocarcinoma ^28, 29^. Recent studies have demonstrated that the bHLH-LZ domain of MAX (MAX*) can be transduced across cell membranes through endocytosis and is able to translocate to the nucleus, suggesting that compounds mimicking the bHLH-LZ domain may be efficacious *in vivo* ^30^. Another recent study extended the efficacy of MYC inhibition as a therapeutic strategy (using Omomyc) in the treatment of human glioma in a mouse model of astrocytoma, human glioblastoma cell lines, and patient-derived tumors *in vitro* and *in vivo* ^31^. Interestingly, general inhibition of MYC activity is tolerated in the mouse, albeit with severely reduced proliferation in the skin, testes, gastrointestinal tract, and hematopoietic lineages ^29^. Remarkably, the proliferation defects were fully reversible, suggesting that anti-MYC therapy could be used to treat human disease since tumor cells often apoptose upon MYC inhibition whereas normal cells simply fail to proliferate.

## Indirectly targeting MYC through BRD4 bromodomain inhibition

Recent findings suggest that MYC promotes gene expression by global transcriptional amplification, although there have been other interpretations of the data ^4, 5, 32, 33^. The transcriptional amplification model proposes that MYC binds to virtually all active promoters in any cells and enhances transcriptional elongation. These studies have established a positive correlation between MYC levels and phosphorylation of serine-2 (S2) on the carboxy-terminal domain (CTD) of RNA Pol II, which is linked to transcriptional elongation. Phosphorylation of S2 on the CTD is catalyzed by P-TEFb (positive transcription elongation factor b), which can be activated by binding to the bromodomain protein BRD4 ^34^. Bromodomains bind to acetylated lysines (Ac-K) on histones and other proteins, and the binding of BRD4 to P-TEFb results in recruitment to promoters and productive transcriptional elongation ^34– 37^. BRD4 is a member of the BET family of proteins and by itself is a key mediator of an aggressive squamous cancer, NUT midline carcinoma ^38^. Small-molecule screens have identified compounds that inhibit the binding of the BRD4 bromodomain to Ac-K ^39, 40^. The most extensively characterized compound developed for this purpose is JQ1, a powerful inhibitor of BRD4 ^39^. JQ1 binds to the Ac-K-binding site of BET bromodomains and effectively displaces BRD4 from chromatin, preventing transcriptional elongation. Treatment with JQ1 results in cell differentiation of NUT cells and attenuates growth of BRD4-dependent carcinomas *in vivo*. Efficacy of JQ1 in a number of myeloid-derived tumors, such as acute myeloid leukemia (AML) and multiple myeloma, has been demonstrated. Notably, these studies have revealed that the effect of JQ1 on tumor regression appears to be specifically mediated by downregulation of MYC itself, its downstream targets, and inflammatory signals ^12, 16, 41, 42^. The link between JQ1 and MYC expression is not totally clear but may involve the dependence of MYC on multiple enhancers and ‘super-enhancers’ that are highly dependent on BRD4 ^43^. These findings have led to a number of potential combination therapies in conjunction with JQ1 that synergistically result in tumor regression. These therapies include indirectly targeting MYC in combination with the PI3K pathway, mechanistic target of rapamycin (mTOR), or histone deacetylases (HDACs) for the treatment of T-cell acute lymphoblastic leukemia, pancreatic ductal adenocarcinoma, and osteosarcoma, respectively ^44– 46^. BET inhibitors have also been shown to induce apoptosis of osteosarcoma cells independently of MYC downregulation and display synergistic effects when combined with CDK inhibitors, indicating that this strategy could be employed in the treatment of osteosarcoma ^47^.

The use of bromodomain-binding compounds has very recently been developed into a new strategy to target BRD4 and subsequently MYC. dBET1 is a novel compound developed to target BRD4 for protein degradation, in contrast to JQ1, which inhibits the bromodomain of BRD4 ^17, 18^. dBET1 is a bivalent compound composed of JQ1 and thalidomide that creates a link between BRD4 and cereblon (CRBN), a component of a cullin-RING ubiquitin ligase that catalyzes proteasomal degradation ^48^. dBET1 is potent and highly specific, targeting BRD2, BRD3, and BRD4 for degradation. As with JQ1, the MYC protein and its transcriptional pathway appear to be the most strongly affected. Treatment with dBET1 produces an improved apoptotic response at lower concentrations in AML and lymphoma cell lines, accompanied by a decrease in MYC levels compared with JQ1. This strategy can be exercised to target a wide variety of proteins that may have no innate enzymatic function as long as high-affinity ligands are available.

## Targeting CDK7 as an indirect inhibitor of MYC

Another very recent study suggests a second indirect approach to target MYC. TFIIH, a complex involved as a basal factor in transcriptional initiation, is composed of a number of proteins, including the catalytic subunit cyclin-dependent kinase 7 (CDK7) ^49, 50^. CDK7 phosphorylates serine-5 (S5) on the CTD of RNA Pol II, which induces transcriptional initiation, production of nascent mRNA, mRNA capping and methylation, and promoter proximal pausing ^51, 52^. THZ1 was developed as a novel covalent inhibitor of CDK7, and its high selectivity for CDK7 results from chemical linkage to a cysteine residue that resides outside of the canonical kinase domain ^53^. Interestingly, THZ1 specifically downregulates MYC in *MYCN*-driven neuroblastomas compared with normal cells, and this effect is attributed to the presence of super-enhancers upstream of the *MYCN* gene ^15^. Although the mechanism accounting for MYC specificity requires further investigation, targeting CDK7 in tumors addicted to super-enhancer-associated transcription factors provides a novel platform for targeting multiple aberrant genes with a single agent. Therapeutically, THZ1 was shown to be highly effective in killing MYC-driven tumors, including neuroblastoma, small cell lung cancer, and triple-negative breast cancer ^15, 54, 55^. Treatment with THZ1 leads to a substantial reduction in tumor volume by suppressing cell proliferation and inducing apoptosis. THZ2, an analog of THZ1, was developed to overcome the instability of THZ1 *in vivo* and demonstrated improved pharmacokinetics with an amended half-life and high potency for CDK7 ^55^. Together, these data provide a rationale for targeting CDK7 in tumors that are dependent on high levels of MYC for transcription.

## Synthetic lethal interactions with MYC

Although MYC itself is difficult to drug, tumor cells often exhibit ‘oncogene addiction’ or changes in gene expression and physiology that make them extremely dependent on a specific oncogenic pathway for growth or survival or both. This dependence theoretically can be exploited to search for a tumor cell’s Achilles heel (that is, pathways that become rate-limiting for the growth/survival of tumor cells but not their normal counterparts). An early study identified AMPK (AMP-dependent kinase) as critical for the survival of cells with high levels of MYC ^56^. Synthetic lethality has also been observed in MYC-overexpressing cells when spliceosome core factors or metabolic pathways are targeted for inhibition ^57, 58^. A more general approach has been taken to uncover new therapeutics for cancer by interrogating the connection between genomic aberrations and response to a wide panel of anti-cancer drugs ^59^. Bioinformatic tools were used to identify a synthetic lethal relationship between MYC overexpression and sensitivity to dasatinib, a multikinase inhibitor. This platform sets a framework for the discovery of novel combination therapies to target MYC-driven tumors.

## Conclusions and Future directions

A large number of direct and indirect MYC inhibitors have been developed in the last decade, and some are more efficacious and specific than others. Although direct inhibitors of MYC, precisely those targeting the interaction between MYC and MAX, are more specific for MYC itself, they target a bHLH-LZ domain conserved between many transcription factors. Chemical inhibition of a domain present in MYC alone would provide a more targeted approach for MYC inhibition. The mechanisms by which indirect inhibitors of MYC, such as JQ1 and THZ1, act remain to be well characterized. Furthermore, additional experimentation is required to determine the efficacy of these compounds in human cancer. Ultimately, it may be necessary to strategically target MYC from a multitude of angles, taking advantage of its well-established role as a master regulator of transcription in cancer cells.
